# Genetic characterization of two fully sequenced multi-drug resistant plasmids pP10164-2 and pP10164-3 from *Leclercia adecarboxylata*

**DOI:** 10.1038/srep33982

**Published:** 2016-09-23

**Authors:** Fengjun Sun, Dongsheng Zhou, Qiang Sun, Wenbo Luo, Yigang Tong, Defu Zhang, Qian Wang, Wei Feng, Weijun Chen, Yahan Fan, Peiyuan Xia

**Affiliations:** 1 Department of Pharmacy, Southwest Hospital, the Third Military Medical University, Chongqing 400038, China; 2 State Key Laboratory of Pathogen and Biosecurity, Beijing Institute of Microbiology and Epidemiology, Beijing 100071, China; 3 Transfusion Department, Southwest Hospital, the Third Military Medical University, Chongqing 400038, China; 4 Beijing Institute of Genomics, Chinese Academy of Sciences, Beijing, 100029, China; 5 College of Food Science and Project Engineering, Bohai University, Jinzhou 121013, China

## Abstract

We previously reported the complete sequence of the resistance plasmid pP10164-NDM, harboring *bla*_NDM_ (conferring carbapenem resistance) and *ble*_MBL_ (conferring bleomycin resistance), which is recovered from a clinical *Leclercia adecarboxylata* isolate P10164 from China. This follow-up work disclosed that there were still two multidrug-resistant (MDR) plasmids pP10164-2 and pP10164-3 coexisting in this strain. pP10164-2 and pP10164-3 were completely sequenced and shown to carry a wealth of resistance genes, which encoded the resistance to at least 10 classes of antibiotics (β-lactams. macrolides, quinolones, aminoglycosides, tetracyclines, amphenicols, quaternary ammonium compounds, sulphonamides, trimethoprim, and rifampicin) and 7 kinds of heavy mental (mercury, silver, copper, nickel, chromate, arsenic, and tellurium). All of these antibiotic resistance genes are associated with mobile elements such as transposons, integrons, and insertion sequence-based transposable units, constituting a total of three novel MDR regions, two in pP10164-2 and the other one in pP10164-3. Coexistence of three resistance plasmids pP10164-NDM, pP10164-2 and pP10164-3 makes *L. adecarboxylata* P10164 tend to become extensively drug-resistant.

*Leclercia adecarboxylata*, which is ubiquitously distributed in nature, is a motile, aerobic member of Enterobacteriaceae, and it shows high degree of phenotypic similarity to *Escherichia coli. L. adecarboxylata* infections are rarely reported in humans, emphasizing the nature of this bacterium as an opportunistic pathogen^1^,^2^,^3^. In most cases, *L. adecarboxylata* is isolated as a pure culture from immunocompromised persons or patients with underlying medical conditions, and its can be occasionally found as a part of polymicrobial cultures in immunocompetent patients suggesting the dependence of this microorganism on co-flora to cause a disease^1^,^2^,^3^. In addition, only two cases of *L. adecarboxylata*-induced monomicrobial infections–without other coinciding pathogens–have also been reported in immunocompetent patients, indicating the relevant isolates may possess unique virulence factors not found in the other reported clinical isolates^4^,^5^. It has been postulated that *L. adecarboxylata* infections are underestimated and have been under-reported for a long time due to the fact of misidentification of this microorganism as *Escherichia coli*^6^.

*L. adecarboxylata* strains are naturally susceptible to all but two β-lactams (see below), azithromycin, tetracyclines, aminoglycosides, quinolones, and amphenicols, but resistant to penicillin G and oxacillin, erythromycin, roxithromycin and clarithromycin, fosfomycin, ketolides, lincosamides, glycopeptides, and rifampicin^6^. There are few reports describing the antimicrobial resistance of *L. adecarboxylata* due to acquisition of foreign resistance gene(s). The SHV-12-mediated cephalosporin resistance^7^, or the carbapenem resistance due to production of carbapenemase KPC-2^8^ or VIM-1^9^ has been observed in clinical *L. adecarboxylata*. In addition, reported are two clinical isolates of multidrug-resistant (MDR) *L. adecarboxylata*, one harboring *bla*_TEM-1_, *bla*_CTX-M-3_, and a class 1 integron cassette array *dfrA12*-*orfF*-*aadA2*^10^, and the other possessing *bla*_SHV-12_, *bla*_DHA-1_-*ampR* and a class 1 integron cassette array *aacA4cr*-*bla*_OXA-1_-*catB3*-*arr3*^11^.

We recently reported a fully sequenced resistance plasmid pP10164-NDM, harboring a total of two resistance genes *bla*_NDM_ (conferring carbapenem resistance) and *ble*_MBL_ (conferring bleomycin resistance), from the clinical *L. adecarboxylata* isolate P10164^12^. Strain P10164 is resistant to β-lactams including carbapenems, quinolones, aminoglycosides, macrolides, fosfomycin, tetracyclines, amphenicols, and trimethoprim/sulfamethoxazole but remained susceptible to tigecycline and polymyxin E. This follow-up study provides the evidence for the presence of two additional resistance plasmids pP10164-2 and pP10164-3 in *L. adecarboxylata* P10164. These two multidrug-resistant (MDR) plasmids were fully sequenced and shown to carry a large amount of antibiotic and heavy metal resistance genes.

## Results and Discussion

### Overview of plasmids pP10164-2 and pP10164-3

**Figure 1:**
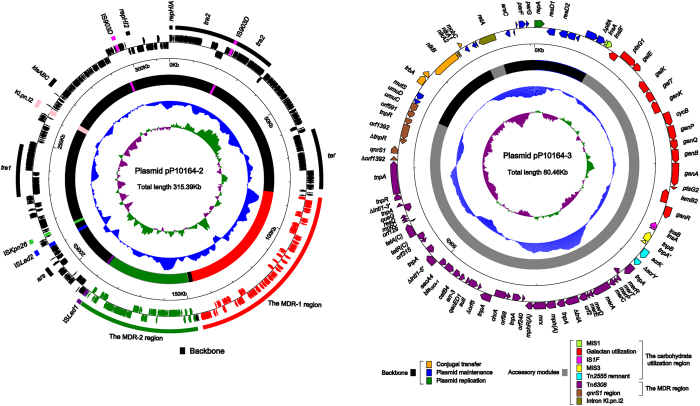
Schematic maps of pP10164-2 (**a**) and pP10164-3 (**b**). Genes are denoted by arrows and colored based on gene function classification. The innermost circle presents GC-Skew [(G − C)/(G + C)] with a window size of 500 bp and a step size of 20 bp. The blue circle presents GC content. Shown also are backbone and accessory module regions.

The complete sequences of pP10164-2 and pP10164-3 were determined from the genomic DNA of strain P10164 by high-throughput shotgun sequencing (the mean sequencing coverages are 79 × and 93 × respectively) and PCR-based gap closing. These two plasmids have circularly closed DNA sequences, 313,395 bp and 80,460 bp in length with mean G + C contents of 47.3% and 54.1%, respectively, and they contain 356 and 91 predicted open reading frames (ORFs) in total, respectively (Fig. 1). The modular structure of each plasmid is discriminated as the backbone with insertion of multiple separate accessory modules.

The pP10164-2 backbone, 205 kb in length, is closely related (97% query coverage and maximum 99% nucleotide identity) to the prototype IncHI2 plasmid R478 from *Serratia marcescens*^13^, and almost identical (100% coverage and 99% identity) to another IncHI2 plasmid pKST313 from *Salmonella enterica* serotype Typhimurium^14^. Located in the pP10164-2 backbone are genes or gene clusters that encode the core IncHI2 plasmid determinants such as *repHIA* and *repHI2* (replication initiation), the *tra1* and *tra2* regions (conjugal transfer), *parAB* and *parM***-***parR* (partition) within *tra2, ter* (tellurium resistance), *klaABC* (plasmid maintenance), and *ars* (arsenic resistance). It has been proposed that the *repHIA* replicon, the essential *trh* (conjugal transfer), *tra*, and *oriT* (origin of transfer) sequences within *tra1* and *tra2*, and the *parAB* partitioning module might represent the minimal IncHI2 determinants^13^.

The pP10164-2 accessory regions, which are dramatically different from R478 and pKST313, are composed of the group IIB1 intron Kl.pn.I2, IS*Kpn26*, two IS*903D* elements, a novel insertion sequence (IS) of IS*3*-family designated IS*Lad1*, a novel IS element of IS*1202* group named IS*Lad2*, and two novel MDR regions designated MDR-1 and MDR-2. The MDR-1 and MDR-2 regions, 61.3 kb and 40.3 kb in length respectively, are adjacent and isolated by a 1.9 kb backbone region composing of two ORFs *orf381* and *∆orf666*.

The pP10164-3 backbone encodes the plasmid replication (*repA*) and maintenance (*parFG* and *umuCD*) functions as well as the residual conjugal transfer determinants (*traA*, mutated *nikAB*, and *mobC*), and overall it exhibits no significant sequence similarity to any known DNA sequences. The deduced replication initiator protein RepA belongs to the Rep_3 superfamily and cannot be assigned into any known incompatibility groups, and it matches various plasmid RepA proteins of unknown incompatibility groups from *Leclercia, Cronobacter* and *Enterobacter* with above 93% amino acid identity.

pP10164-3 is quite unusual because it has a relatively small (19 kb in length) backbone but carries much larger accessory contents including the 2.3 kb Kl.pn.I2 intron and a 37.8 kb region composed of a MDR region and a carbohydrate utilization region. The carbohydrate utilization region is sequentially organized as a mutated sequence of a novel IS*1*-family member (MIS1), a novel 14-gene locus probably accounting for galactan utilization, IS*1F*, a mutated sequence of a novel IS*3*-family member (MIS3), and a Tn*2555* remnant. Both MIS1 and MIS3 cannot be discriminated as intact IS elements because their transposase genes *insB* and *tnpA*, respectively, becomes pseudogenes due to frameshift. The sucrose transposon Tn*2555* from *E. coli* is an IS*26*-based composite transposon that carries the sucrose utilization gene cluster *scrKYABR*, two direct IS*26* copies on its flanks and, sometimes, a third inverted IS*26* copy inside the transposon^15^, while the Tn*2555* remnant from pP10164-3 containing only *scrK* and *∆scrY.*

### The MDR-1 region of pP10164-2

**Figure 2:**
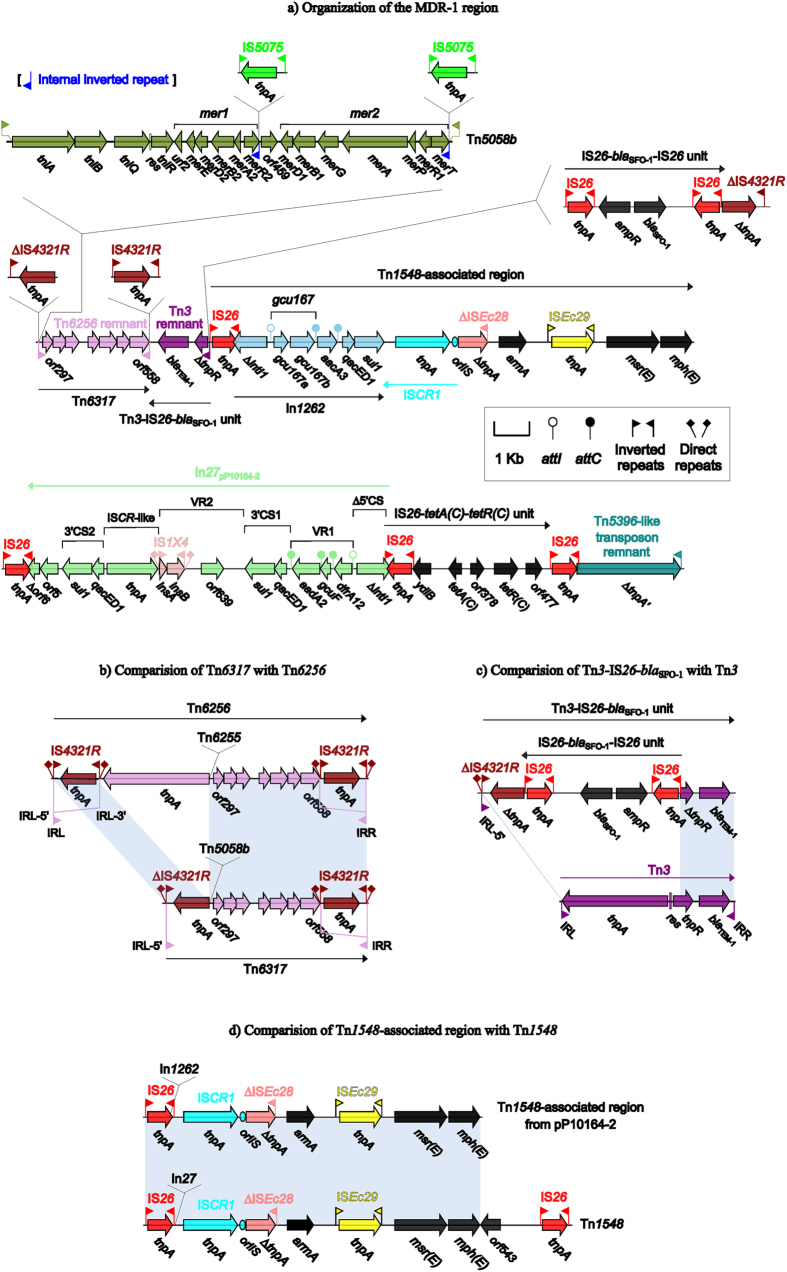
The pP10164-2 MDR-1 region and comparison to related regions. Genes are denoted by arrows and colored based on gene function classification. Shading regions denote regions of homology (>95% nucleotide similarity).

The pP10164-2 MDR-1 region (Fig. 2a) is organized sequentially as a novel Tn*3*-family unit transposon designated Tn*6317*, the Tn*3*-IS*26*-*bla*_SFO-1_ unit, a Tn*1548*-associated region, IS*26*, In*27*_pP10164-2_, the IS*26*-*tetA(C)*-*tetR(C)* unit, IS*26*, and a Tn*5396*-like transposon remnant.

Tn*6317* is generated from the insertion of Tn*5058b* into a backbone remnant of Tn*6256*, a Tn*3*-family Tn*Pa38*-related transposon from clinical *Citrobacter freundii* from Italy^16^. Each of the two 39 bp terminal inverted repeats (IRL: inverted repeat left; IRR: inverted repeat right) of Tn*6317* is disrupted by IS*4321R* into two separate parts (IR-5′ plus IR-3′), which is also observed in Tn*6256*. It seems that the Tn*5058b* insertion is accompanied by not only the truncation of IS*4321R* but also the loss of downstream IRL-3′ and the core transposition module *tnpA* (transposase) at the 5′ region of Tn*6317* relative to Tn*6256* (Fig. 2b). Tn*5058b* is composed of a Tn*5053*-family core transposition module *tniA* (transposase)-*tniB* (ATP-binding protein)-*tniQ* (transposition auxiliary protein)-*res* (resolution site)-*tniR* (serine resolvase) and two mercury resistance gene clusters named *mer1* and *mer2*, which is delimited by terminal 25 bp IRL and IRR.Tn*5058b* differs from the prototype Tn*5058* (accession number Y17897) from *Pseudomonas* sp. ED23-33 by the insertion of IS*5075* into each of the two internal inverted repeats IIR_*merT*_ and IIR_*merR2*_. The IS*1111*-family IS*4321* and its close derivative IS*5075* are known to target the terminal inverted repeats of the Tn*21*-subgroup transposons of Tn*3* family^17^.

The Tn*3*-IS*26*-*bla*_SFO-1_ unit is likely derived from a precursor Tn*3* [IRL-*tnpA*-*res*-*tnpR* (resolvase)-*bla*_TEM-1_-IRR], which has undergone at least two major evolutionary events (Fig. 2c): i) The disruption of the 38 bp IRL of Tn*3* into IRL-5′ and IRL-3′ by IS*4321R*; and then ii) the insertion of the IS*26*-*bla*_SFO-1_-IS*26* unit (which is known to be transposable among plasmids^18^) upstream of IS*4321R*, leading to the truncation of IS*4321R*, the loss of IRL-3′-*tnpA*-*res* of Tn*3*, and the truncation of *tnpR* of Tn*3*. The connection of Tn*3*-IS*26*-*bla*_SFO-1_ with Tn*6317* orientated in opposite directions likely results in the loss of the IRR of Tn*3*, making Tn*3*-IS*26*-*bla*_SFO-1_ cannot to be discriminated as a transposon due to the absence of one of the paired IRL/IRR routinely bracketing at both ends. Both *bla*_TEM-1_ and *bla*_SFO-1_ encode class A β-lactamases, whose activity can be inhibited by clavulanic acid. TEM-1 is able to hydrolyze penicillins but not extended-spectrum cephalosporins; by contrast, SFO-1 exhibits significant hydrolytic activity against both penicillins and extended-spectrum cephalosporins, but it has no detectable activity against carbapenems and cephamycins^19^. The *bla*_SFO-1_ expression is inducible, which is regulated by the transcriptional activator encoded by *ampR* that is inversely orientated upstream of *bla*_SFO-1_^20^.

Tn*1548* is an IS*26*-based composite transposon from the *C. freundii* plasmid pCTX-M3 and displays a modular structure IS*26*-In*27*-IS*CR1*-∆IS*Ec28*-*armA*-IS*Ec29- msr(E)*-*mph(E)*-*orf543*-IS*26*^21^,^22^. Notably, Tn*1548* lacks the paired short direct repeats (DRs), which represent the target site duplication signals routinely bracketing at both ends of a composite transposon. Tn*1548* and various Tn*1548*-asscoiated elements (with insertion of different class 1 integrons or integron-like sequences between IS*26* and IS*CR1*) are thought to promote the dissemination of the aminoglycoside resistance gene *armA*, the macrolide resistance operon *msr(E)*-*mph(E)*, and other classes of antibiotic resistance genes within the inserted integrons^23^. The Tn*1548*-asscoiated region from pP10164-2 differs from Tn*1548* by the replacement of In*27* by a novel class 1 integron named In*1262*, and the deletion of *orf543*-IS*26* originally at the 3′ region of Tn*1548* (Fig. 2d). The connection of immediately upstream IS*26* and immediately downstream IS*CR1* with In*1262* leads to the loss of two terminal 25 bp inverted repeats (IRi: inverted repeat initial; IRt: inverted repeat terminal) and the truncation of *intI1* (integrase) occurred for this integron. In*1262* carries two gene cassettes *gcu167* and *aacA3* (aminoglycoside resistance):*attC*_*aacA3*_. The novel gene cassette *gcu167* of unknown function contains two consecutive ORFs *gcu167a* (putative nudix hydrolase) and *gcu167b* (putative nucleotidase), followed by a single *attC*_*gcu167*_ site.

In*27*_pP10164-2_ resembles a complex class 1 integron, whose modular structure can be generally divided sequentially into 5′-conserved segment [5′CS: *intl1*-*attI*], variable region 1 (VR1), the first copy of 3′-conserved segment [3′CS1: *qacE∆1* (quaternary ammonium compound resistance)-*sul1* (sulfonamide resistance)], common region IS*CR1*, VR2, and the second copy of 3′CS (3′CS2), bordered by terminal 25 bp IRi and IRt^24^. In*27*_pP10164-2_ comprises *∆*5′CS (*∆intl1*-*attI*), VR1 [three sequentially arranged gene cassette*s: dfrA12* (trimethoprim resistance):*attC*_*dfrA12*_, *gcuF* (unknown function):*attC*_*gcuF*_, and *aadA2* (aminoglycoside resistance):*attC*_*aadA2*_], 3′CS1, VR2 [*orf639* (putative β-lactamase)-IS*1* × 4], an IS*CR*-like element, 3′CS2, *orf5*, and *∆orf6; ∆*5′CS and *∆orf6* are in truncated formats and IRi and IRt are absent, which is likely resulted from the connection of IS*26* at both ends of In*27*_pP10164-2_. The common region IS*CR1*, which is commonly located between 3′CS1 and VR2 of a typical complex class 1 integron, is not found in In*27*_pP10164-2_, but a 1.4 kb element (which encodes a putative protein with 80% amino acid sequence similarity to the IS*CR20* transposase but lacks the *oriIS* element characteristic of IS*CR*s) is found between VR2 and 3′CS2 of In*27*_pP10164-2_.

IS*26*-*tetA(C)*-*tetR(C)* represents a putative mobile unit carrying a tetracycline resistance module *tetA(C)* (tetracycline efflux protein)-*tetR(C)* (transcriptional repressor of *tetA*); moreover, similar genetic elements are found in various plasmids such as the IncN1 plasmid N3^25^, the IncHI2 plasmids pMRVIM0813 (accession number KP975077), pSTm-A54650 (LK056646) and pKST313^14^, and the partially sequenced plasmid pQKp274H^26^. Located at the 3′ terminus of the MDR-1 region is a 2.9 kb transposon remnant, which contains the 38 bp IRL and a pseudogene of *tnpA* (with truncation and frameshift) and shows 96% nucleotide sequence identity to the Tn*3*-family transposon Tn*5396*^27^.

At least 6 copies of IS*26* are found in the MDR-1 region and can be arbitrarily assigned into the four structures IS*26*-In*1262-*IS*CR1*-∆IS*Ec28*-*armA*-IS*Ec29-msr(E)*-*mph(E)-*IS*26*, IS*26*- *bla*_SFO-1_-IS*26*, IS*26*-In*27*_pP10164-2_*-*IS*26*, and IS*26*-*ydiB*-*tetA(C)*-*orf378*-*tetR(C)*-*orf477 -*IS*26*. Each of them contains two terminally flanking IS*26* elements but cannot be annotated as a composite transposon, because the paired DR sequences are not identified. The common component IS*26* would act as an adaptor to mediate massive recombination and transposition events^28^,^29^, facilitating the assembly of the MDR-1 region with a very complex mosaic structure.

### The MDR-2 region of pP10164-2

**Figure 3:**
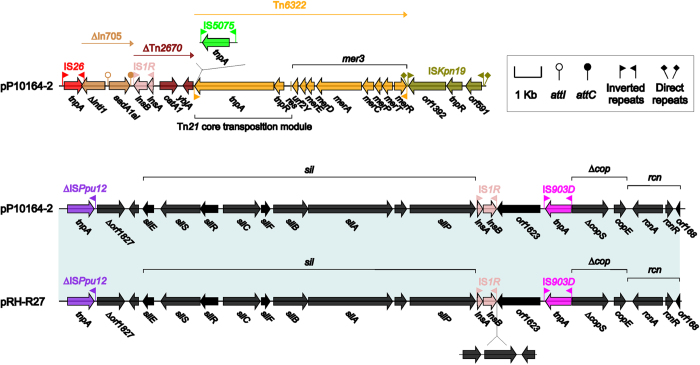
The pP10164-2 MDR-2 region and comparison to related region. Genes are denoted by arrows and colored based on gene function classification. Shading regions denote regions of homology (>95% nucleotide similarity).

The pP10164-2 MDR-2 region (Fig. 3) is mainly composed of IS*26*, ∆In*705*, ∆Tn*2670*, a novel Tn*3*-family unit transposon designated Tn6*322*, IS*Kpn19*, ∆IS*Ppu12* lacking IRL, *sil*, IS*1R*, IS*903D*, ∆*cop* and *rcn* in order of their priority. ∆In*705* contains *∆*5′CS (*∆intI1*-*attI*, truncated by connection of IS*26* upstream of ∆In*705*) and a single gene cassette *aadA1ai* (aminoglycoside resistance):*attC*_*aadA1ai*_. The *aadA1ai* gene is a derivative of the prototype *aadA1* gene (accession number X12870), displaying the Val5Met amino acid substitution.

Tn*2670* is an IS*1*-based composite transposon, which is composed of a backbone region with Tn*21* inserted within it^30^ and originally found in the MDR plasmid R100 (accession number AP000342) from *Shigella flexneri*. The In*2670* backbone consists of two IS*1* elements flanking a 1.5 kb central region that harbors the amphenicol resistance gene *catA1* and the *ybjA* gene encoding putative acetyl transferase^30^. ∆Tn*2670* from the pP10164-2 MDR-2 region resembles the In*2670* backbone but lacks the right terminal IS*1* and, notably, similar structures are found in other IncHI2 plasmids such as pRH-R27^31^ and in the chromosomally located resistance island AbaR1 and its derivatives from *Acinetobacter baumannii*^32^.

Tn6*322* is composed of the Tn*21* core transposition module *tnpAR*-*res*^33^ together with a novel mercury resistance gene cluster designated *mer3*, and the *mer3* region differs dramatically (92% coverage and maximum 86% nucleotide identity) from the *mer* locus from Tn*21*, indicating the capture of *mer3* by the Tn*21* core transposition module during the genesis of Tn6*322*. The *mer3* region is mostly similar (100% coverage and maximum 96% nucleotide identity) to the counterpart of the *Enterobacter cloaca*e transposon Tn*6005* belonging to the Tn*5036*/Tn*3926* subgroup of Tn*3* family^34^. Tn6*322* is flanked of 38 bp IRL/IRR resembling those of Tn*21*: the IRR is intact, while the IRL shows the insertion of IS*5075*.

Silver and copper compounds are used as antimicrobial agents in hospitals, and the relevant resistance determinants could serve as hygienic fitness factors and thus improve bacterial survival in hospital environments. In R478, the silver and copper resistance gene clusters, called *sil* and *cop* respectively, are located adjacently and associated with an upstream Tn*7*-like core transposition module *tnsABCD*. Similar *tnsABCD-sil*-*cop* structures are widely found in IncHI2 plasmids such as pMRVIM0813 (accession number KP975077), pSTm-A54650 (LK056646), pKST313^14^ and pRH-R27^31^, although considerable variations in both genetic content and nucleotide sequence are observed among different plasmids. Similarly, a multi-heavy metal resistance region IS*Kpn19*-∆IS*Ppu12*-*sil*-IS*1R*-*orf1623*-*∆cop*-*rcn* is found in the pP10164-2 MDR-2 region: compared with the prototype *tnsABCD-sil*-*cop* structure, IS*Kpn19*-∆IS*Ppu12* replaces *tnsABCD*, the insertion of IS*1R*-*orf1623* (putative metal-dependent hydrolase)-IS*903D* between *sil* and *cop* marked the truncation of *cop* into *∆copS*-*copE*, and a *rcn* locus (encoding the RcnA efflux pump responsible for nickel/cobalt detoxification and the *rcnA* repressor RcnR) is added immediately downstream of *∆copS*-*copE*. Notably, the IncHI2 plasmid pRH-R27^31^ carries a very similar structure from ∆IS*Ppu12* to *rcn* with further insertion of a fragment composed of three hypothetical ORFs between IS*1R* and *orf1623*^31^. The MDR-2 region of pP10164-2 and the corresponding MDR region of pRH-R27^31^ are genetically related and might share a much more recent ancestor, although they contains dramatically different sets of resistance genes upstream of the ∆IS*Ppu12* to *rcn* region.

### The MDR region of pP10164-3

**Figure 4:**
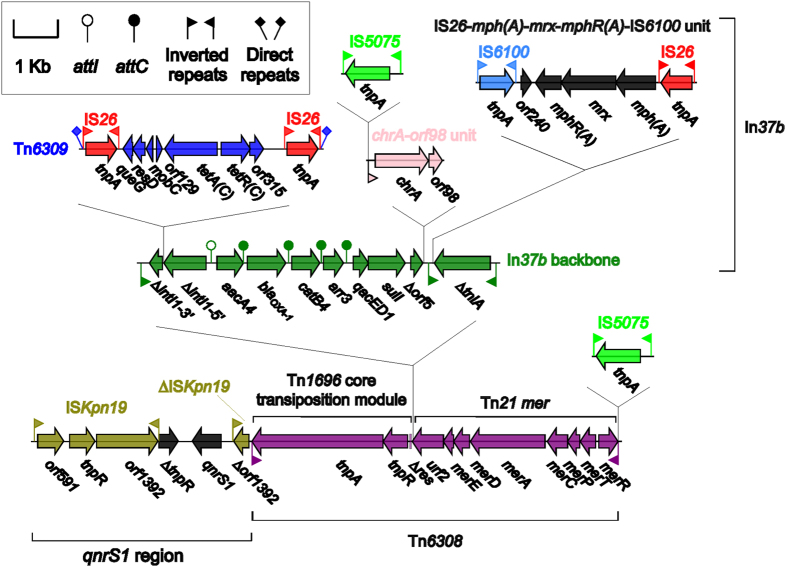
The pP10164-3 MDR region. Genes are denoted by arrows and colored based on gene function classification.

The pP10164-3 MDR region (Fig. 4) is 35.5 kb in length and can be divided into two components, namely a 4.9 kb *qnrS1* (quinolone resistance) region and a novel Tn*3*-family unit transposon designated Tn*6308*. The *qnrS1* genetic platform ∆IS*Ecl2*-*qnrS1*- *∆tnpR* (truncated Tn*3*-family resolvase)-IS*Kpn19* is widely found in resistance plasmids from *Enterobacteriaceae* species^35^. Replacement of the 5′ terminal ∆IS*Ecl2* by ∆IS*Kpn19* generates a novel *qnrS1* region ∆IS*Kpn19*-*qnrS1*-*∆tnpR*-IS*Kpn19* as observed in the pP10164-3 MDR region.

The Tn*6308* backbone is a hybrid of the core transposition module *tnpAR*-*res* of Tn*1696* and the *mer* region of Tn*21*, and it is bordered by the intact 39 bp IRL and the IS*5075-*disrupted IRR at both ends in the absence of DRs. Tn*1696* and Tn*21* are both the members of the Tn*21* subgroup of Tn*3* family, but they have independent histories and origins with limited nucleotide sequence similarity (79 to 96%) between corresponding backbone genes^36^. The *res* site, originally 120 bp in length, is truncated into an 83 bp remnant in Tn*6308* due to the insertion of a class 1 integron In*37b*. Notably, all the three novel Tn*3*-family transposons Tn*6317*, Tn6*322*, and Tn*6308* identified in this work have undergone at least two evolutionary events after their initial transposition into pP10164-2 or pP10164-3: i) the disruption of one or both terminal IR sequences by insertion of IS*5075* or IS*4321R*, making them deficient in further mobilization; and ii) the removal of target site duplication signals, making them lack of terminal DR sequences.

**Figure 5:**
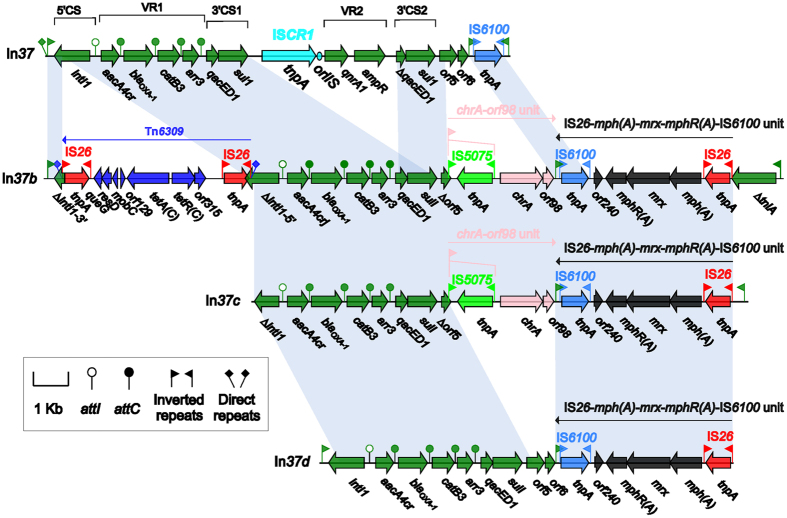
Comparison of In*37b* with its derivatives. Genes are denoted by arrows and colored based on gene function classification. Shading regions denote regions of homology (>95% nucleotide similarity).

In*37b* from pP10164-3, In*37c* from the *C. freundii* plasmid p112298-KPC^37^ and In*37d* from the *Aeromonas* plasmid pP2G1^38^ are all derivatives of the typical complex class 1 integron In*37* from *Escherichia coli*^39^ (Fig. 5). In*37* is sequentially organized as 5′CS, VR1 [*aacA4cr* (quinolone and aminoglycoside resistance):*attC*_*aacA4cr*_], *bla*_OXA-1_ (β-lactam resistance):*attC*_*bla*OXA-1_, *catB3* (amphenicol resistance):*attC*_*catB3*_, and *arr3* (rifampicin resistance):*attC*_*arr3*_], 3′CS1, IS*CR1*, VR2 [*qnrA1* (quinolone resistance) and *ampR* (LysR-family regulator)], 3′CS2, and *orf5*-*orf6*-IS*6100*, which is bracketed by 25 bp IRi/IRt and associated 5 bp DRs^39^.

Compared to In*37*, In*37b* has undergone the insertion of Tn*6309* into *intI1*, the loss of IS*CR1*-VR2-3′CS2, the truncation of *orf5*-*orf6* into ∆*orf5* due to the insertion of the chromate resistance unit IRL_*chrA*_-*chrA*-*orf98*^40^ (the 38 bp IRL_*chrA*_ is further disrupted by IS*5075*), and the replacement of IS*6100* by the macrolide resistance unit IS*26*-*mph(A)*-*mrx*-*mphR(A)*-IS*6100*^40^ followed by *∆tniA*_Tn*21*_ (Fig. 5). Tn*6309* is an IS*26*-based composite transposon containing the tetracycline resistance module *tetA(C)*-*tetR(C)*; although just being named in this work, Tn*6309* has been found in the genomic island Sm1-MDRGI from *Stenotrophomonas maltophilia*^41^ and also in the three sequenced plasmids pB3 from P*seudomonas* sp. GFP1^42^, pKAZ3 from an uncultured bacterium^43^ and pNDM-116-14 (accession number LN831184) from *Vibrio cholerae*. The absence of IRi and *∆tniA*_Tn*21*_ and the truncation of *intI1* are observed but there is no insertion of Tn*6309* in In*37c* compared to In*37b* (Fig. 5). Insertion of Tn*6309* and IRL_*chrA*_-*chrA*-*orf98* are not found in In*37d*, leaving *intI1* and *orf5*-*orf6* intact, but *∆tniA*_Tn*21*_-IRt is absent from In*37d* compared to In*37b* (Fig. 5). The above observations indicate that extensive recombination and transposition events have occurred during derivation of In*37*, In*37b*, In*37c* and In*37d* from an In*37*-like precursor, making them to integrate different sets of additional resistance genes, but the core resistance gene cassette array *aacA4cr*-*bla*_OXA-1_-*catB3*-*arr3* is shared by these integrons.

### Concluding remarks

This is the first report of detection of MDR plasmids and determination of their complete sequences in *L. adecarboxylata*. Coexistence of three resistance plasmids pP10164-NDM, pP10164-2 and pP10164-3 makes *L. adecarboxylata* P10164 tend to become extensively drug-resistant. This bacterial species may serve as a potential reservoir of antimicrobial resistance genes in clinical settings. Data presented here would promote us to gain deeper understanding of plasmid-mediated mechanisms of drug resistance in *L. adecarboxylata*. Prevalence of the resistance plasmids pP10164-NDM, pP10164-2 and pP10164-3 in *L. adecarboxylata* and other bacterial species from the clinical settings cultures especially those from immunocompromised patients needs to be elucidated.

## Methods

Bacterial genomic DNA were isolated by classical phenol/chloroform method followed by diethyl ether removal of polysaccharides that contaminate genomic DNA^44^, and then sequenced with a paired-end library with an average insert size of 500 bp and a mate-pair library with average insert size of 5,000 bp, using HiSeq 2500 sequencer (Illumina, CA, USA). In order to get complete plasmid sequences, the contigs were assembled with Velvet, and the gaps were filled through combinatorial PCR and Sanger sequencing on ABI 3730 Sequencer (LifeTechnologies, CA, USA).

The open reading frames and pseudogenes were predicted with GeneMarkS™ (http://topaz.gatech.edu/GeneMark), RAST (http://rast.nmpdr.org/), and Prodigal (http://compbio.ornl.gov/prodigal), and further annotated by BLASTP and BLASTN against UniProtKB/Swiss-Prot (http://web.expasy.org/docs/swiss-prot_guideline.html) and NCBI NR databases.

Annotation of resistance genes, mobile elements and other gene futures was based on the relevant databases including CARD (http://arpcard.mcmaster.ca), BacMet (http://bacmet.biomedicine.gu.se/), β-lactamases Database (http://www.ncbi.nlm.nih.gov/pathogens/submit_beta_lactamase), ISfinder (https://www-is.biotoul.fr/), ISCR Elements Databases (http://medicine.cf.ac.uk/infect-immun/research/infection/antibacterial-agents/iscr-elements), INTEGRALL (http://integrall.bio.ua.pt/), Tn Number Registry (http://www.ucl.ac.uk/eastman/research/departments/microbial-diseases/tn), and Group II Introns Databases (http://webapps2.ucalgary.ca/~groupii/blast.html). Sequence comparison was performed with BLASTN and CLUSTALW, and gene organization diagrams were drawn with Inkscape (https://inkscape.org). The complete sequences of pP10164-2 and pP10164-3 were submitted to GenBank under accession numbers KX710093 and KX710094, respectively.

## Additional Information

**How to cite this article**: Sun, F. *et al*. Genetic characterization of two fully sequenced multi-drug resistant plasmids pP10164-2 and pP10164-3 from *Leclercia adecarboxylata. Sci. Rep.* **6**, 33982; doi: 10.1038/srep33982 (2016).
